# Modulation of solute diffusivity in brain tissue as a novel mechanism of transcranial direct current stimulation (tDCS)

**DOI:** 10.1038/s41598-020-75460-4

**Published:** 2020-10-28

**Authors:** Yifan Xia, Wasem Khalid, Zhaokai Yin, Guangyao Huang, Marom Bikson, Bingmei M. Fu

**Affiliations:** Department of Biomedical Engineering, The City College of the City University of New York, 160 Convent Ave, New York, NY 10031 USA

**Keywords:** Biophysics, Neuroscience, Physiology, Engineering

## Abstract

The breadth of brain disorders and functions reported responsive to transcranial direct current stimulation (tDCS) suggests a generalizable mechanism of action. Prior efforts characterized its cellular targets including neuron, glia and endothelial cells. We propose tDCS also modulates the substance transport in brain tissue. High resolution multiphoton microscopy imaged the spread across rat brain tissue of fluorescently-labeled solutes injected through the carotid artery after tDCS. The effective solute diffusion coefficient of brain tissue (D_eff_) was determined from the spatio-temporal solute concentration profiles using an unsteady diffusion transport model. 5–10 min post 20 min–1 mA tDCS, D_eff_ increased by ~ 10% for a small solute, sodium fluorescein, and ~ 120% for larger solutes, BSA and Dex-70k. All increases in D_eff_ returned to the control level 25–30 min post tDCS. A mathematical model for D_eff_ in the extracelluar space (ECS) further predicts that this dose of tDCS increases D_eff_ by transiently enhancing the brain ECS gap spacing by ~ 1.5-fold and accordingly reducing the extracellular matrix density. The cascades leading ECS modulation and its impact on excitability, synaptic function, plasticity, and brain clearance require further study. Modulation of solute diffusivity and ECS could explain diverse outcomes of tDCS and suggest novel therapeutic strategies.

## Introduction

Transcranial direct current stimulation (tDCS) generates static electric fields in the brain leading to lasting changes in brain function^1–4^. The breadth of applications tDCS is investigated for^5–8^ suggests a mechanism of action that is on the one hand generalizable^9–11^ while on the other hand tunable to specific outcomes^12–14^. As with other forms of neuromodulation^15,16^, research on tDCS cellular mechanisms has focused on characterizing which neuronal elements are activated^9,17–19^, which has been extended to consider additional cells types notably glial^20,21^ and endothelial cells^22,23^.

The wall of cerebral microvessels is named the blood–brain barrier (BBB) which tightly regulates the brain micro-environment including metabolic capacity and clearance. Being a protecting barrier, the BBB consists of endothelial cells (ECs) with tight junctions between adjacent ECs, which are wrapped by the basement membrane (BM), pericytes and astrocyte foot processes^24–26^. tDCS modulates brain vascular function^9,27–30^ and nitric oxide (NO) signaling^31^ including in patients with Alzheimer’s disease^32^. Cancel et al.^22^ showed that direct current stimulation can modulate hydraulic conductivity of an in vitro BBB model through tight-junction electro-osmosis. Shin et al.^23^ demonstrated that tDCS transiently increases the BBB permeability (P) in rat brain^22,23^ through activation of nitric oxide synthase (NOS).

Brain parenchyma is essentially composed of two regions: cellular elements (neurons and glial cells), and the gaps between these elements, which is known as the extracellular space (ECS)^33–35^. Typically occupying ~ 20% of the total brain tissue volume^34,36,37^, the brain ECS contains interstitial fluid with ions and negatively charged extracellular matrix (ECM)^38,39^. The ECS is a dynamic regulator for the transport of extracellular molecules^37,40,41^, playing a crucial roles in neural growth, excitability, signaling, and plasticity^42,43^. The objective of the current study is to test if tDCS modulates the ECS, as measurable by substance transport in brain tissue, and as distinguishable from additional effects increasing the BBB permeability.

We used a small solute, sodium fluorescein (MW 376), and two large solutes, BSA (bovine serum album, MW ~ 69k) with negative charge (charge number − 19) and Dex-70k with no charge as the representative test substances and quantified their effective diffusion coefficients D_eff_ in rat brain tissue under control and in response to tDCS treatments. D_eff_ is a quantitative indicator for substance transport in porous media, such as brain tissue. To do this, we employed our newly developed non-invasive multiphoton microscopy^44^ to both confirm changes in BBB permeability P^23^ and test D_eff_ under control and after tDCS treatment. The solution with the fluorescently labeled solutes was injected into the rat cerebral circulation via the ipsilateral carotid artery. Simultaneously, the 3-D images of a post-capillary venule and its surrounding area in the rat brain tissue 100–200 μm below the pia mater were collected by laser scanning multiphoton microscopy. The P and D_eff_ were determined from the collected dye spreading images. Specifically, D_eff_ was estimated from the spatio-temporal solute concentration (fluorescence intensity) profiles using an unsteady diffusion transport model.

We report tDCS transiently enhance the effective solute diffusion coefficient D_eff_ in rat brain tissue_._ Based on our measurements for D_eff_ of various sized solutes and the predicted width of the brain ECS^45^, a model for the restricted diffusion of a solute in a slit filled with fiber matrix predicted that tDCS increases D_eff_ by transiently enhancing the width of the brain ECS and reducing ECM density accordingly. Our results thus revealed a new motion of action of tDCS, in parallel to direct neuronal or glial stimulation and enhancement of the BBB permeability. By modulating the ECS, DCS may achieve its therapeutic effect by increasing metabolic capacity and brain clearance mechanisms. Our findings also suggest that tDCS can be used as a non-invasive, tolerated, and low-cost approach for the enhancement of the brain drug delivery, especially macromolecules, delivered through the BBB^23^ or CSF^46^.

## Results

A customized high-resolution multiphoton microscopy system was used to image the spread across rat brain tissue of fluorescently-labeled solutes following their injection through the carotid artery in response to tDCS or under control (not stimulation) conditions (see “Methods” section). We assessed both BBB permeability (P) and the effective solute diffusion coefficient of brain tissue (D_eff_) at two time points (~ 5 min and ~ 25 min) after tDCS.

### Effects of tDCS on the BBB solute permeability (P)

Table 1 summarizes the measured BBB permeability P to various sized solutes under control and the corrections due to the influence from the residue red blood cells (RBCs) in the fluorescent solution perfused microvessel, free dye in the solution of the FITC-conjugated solutes and the solvent drag from the water permeability (hydraulic conductivity) of the microvessel. It shows that P to the small molecule, sodium fluorescein (NaFl, MW = 376), is about 13 and 15 folds of that to the large molecules, Dex-70k and BSA (MW ~ 69k), which have similar size but one is neutral and another carries negative charge. The net charge number of FITC-BSA is − 19^47^. Since both the endothelial surface glycocalyx and the extracellular matrix in the basement membrane of the BBB carry negative charge^48^, P to negatively charged BSA (0.99 ± 0.11 × 10^−7^ cm/s) is less than that to neutral Dex-70k (1.22 ± 0.05 × 10^−7^ cm/s, *p* = 0.03). Those values for the control P of the BBB were used in Eq. (6) to predict the spatio-temporal solute concentration profiles in the brain tissue. Matching the predicted with the measured profiles allowed us to determine the effective solute brain tissue diffusion coefficient D_eff_ under control conditions.

**Table 1:** Measured and corrected control permeability.

Solute	n	Vessel radius (μm)	P (measured) (× 10^−7^ cm/s)	P (corrected for RBCs) (× 10^−7^ cm/s)	P (corrected for RBCs and free dye) (× 10^−7^ cm/s)	P (corrected for RBCs, free dye, and solvent drag) (× 10^−7^ cm/s)
Sodium fluorescein	8	10.2 ± 0.1	17.51 ± 2.34	15.77 ± 2.11	15.77 ± 2.11	15.77 ± 2.11 (L_p,control_ = 2 × 10^−9^)
FITC-BSA (− 19)	10	12.3 ± 0.5	1.13 ± 0.12	1.02 ± 0.11	0.99 ± 0.11	0.99 ± 0.11 (L_p,control_ = 2 × 10^−9^)
FITC-Dex-70k	11	11.5 ± 0.1	1.38 ± 0.06	1.24 ± 0.05	1.22 ± 0.05	1.22 ± 0.05 (L_p,control_ = 2 × 10^−9^)

Values are mean ± SE. n = number of vessels. Hydraulic conductivity L_p_ in cm/s/cm H_2_O. Control L_p,control_ is from Kimura et al. (1993).

Table 2 demonstrates the effects of tDCS on P to various sized solutes. Due to the limitation of our current technique, we can only determine the P post tDCS treatment. We found that 5–10 min post 20 min–1 mA tDCS significantly increased P to NaFl, BSA and Dex-70k to 13.2-fold, 104.6-fold, and 86.7-fold (*p* < 0.01), respectively. All the increased P by tDCS returned to their control values in 25–30 min (*p* > 0.1). These values of P post tDCS treatment were inserted in Eq. (6) to find the corresponding D_eff_ 5–10 min and 25–30 min post tDCS treatment.

**Table 2:** tDCS modulated permeability.

Solute	$\frac{{{\text{P5 } - \text{ 10 }}\,{\min}\,{\text{post tDCS }}}}{{{\text{P}}_{{{\text{control}}}} { }}}$	$\frac{{{\text{P 25 } - \text{ 30}}\,{\min}\,{\text{post tDCS }}}}{{{\text{P}}_{{{\text{control}}}} { }}}$
Sodium fluorescein	13.2 ± 2.1 (n = 5)	1.0 ± 0.2 (n = 6)
FITC-BSA (− 19)	104.6 ± 19.7 (n = 7)	1.2 ± 0.3 (n = 8)
FITC-Dex-70k	86.7 ± 9.8 (n = 7)	1.3 ± 0.4 (n = 7)

Values are mean ± SE. n = number of vessels.

### Effects of tDCS on solute transport in brain tissue (D_eff_)

**Figure 1:**
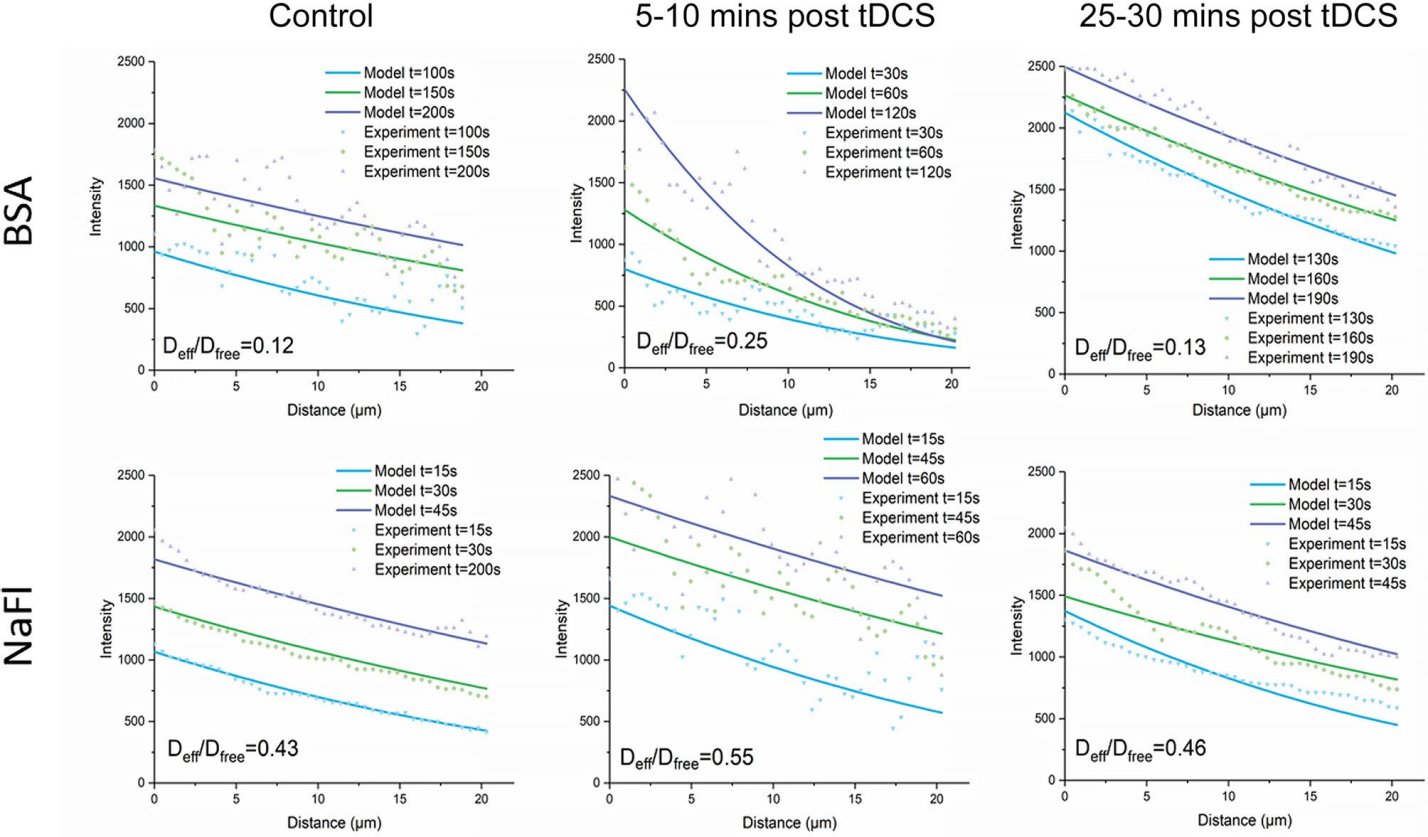
Effects of tDCS on solute transport in the brain tissue (D_eff_). The dots are the measured spatial–temporal intensity profiles of the spreading fluorescently labeled solutes in the brain tissue and the smooth lines are the model predictions with the best fitting values of D_eff_/D_free_, the ratio of the solute effective diffusion coefficient to its free diffusion coefficient. The top panel is for a large solute, FITC-BSA and the bottom panel for a small solute, sodium fluorescein (NaFl), under control, 5–10 min, and 25–30 min post tDCS, respectively.

**Figure 2:**
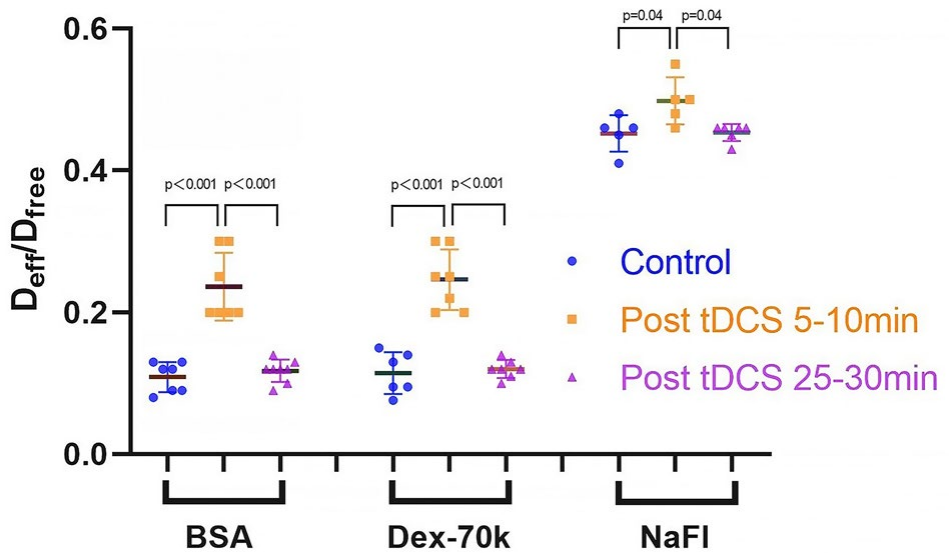
Comparison of the effects of tDCS on solute transport in the brain tissue for various sized solutes. D_eff_/D_free_ for the large solutes, negatively charged FITC-BSA and neutral FITC-Dex-70k and for a small solute, sodium fluorescein (NaFl), under control, 5–10 min and 25–30 min post tDCS.

Figure 1 demonstrates the effects of tDCS on solute transport in brain tissue, which is quantified by the effective solute diffusion coefficient in brain tissue D_eff_. The upper panel shows the effect of tDCS on D_eff_ of the large solute, BSA, and the bottom panel shows that on D_eff_ of the small solute, sodium fluorescein. The effect of tDCS on D_eff_ of neutral Dex-70k is similar to that of negatively charged BSA with the same size. In each plot shown in Fig. 1, the colored dots are the measured spatio-temporal solute concentration profiles in the brain tissue surrounding an individual microvessel (see Fig. 4C,D), while the colored lines are the best matching profiles predicted by an unsteady diffusion solute transport model (Eqs. 5–8) when the D_eff_ is properly chosen. Under control, D_eff_/D_free_ is 0.12 for BSA in the surrounding brain tissue of one microvessel shown in the upper left plot. D_free_ is the solute diffusion coefficient in aqueous solution (e.g. interstitial fluid) at 37 °C (see Table 3). 5–10 min post tDCS treatment, D_eff_/D_free_ becomes 0.25 for BSA in the surrounding tissue of another microvessel shown in the upper middle plot. After 25–30 min post tDCS treatment, D_eff_/D_free_ returns to 0.13 for BSA in the surrounding tissue of a different microvessel shown in the upper right plot. The bottom panel shows the effect of tDCS on D_eff_ of sodium fluorescein (NaFl). Since NaFl (Stokes radius ~ 0.45 nm) is much smaller than BSA (Stokes radius ~ 3.5 nm) and Dex-70k (Stokes radius ~ 3.6 nm), not only is its D_free_ much larger, about one order of magnitude higher of those of BSA and Dex-70k, but also the relative transport in the brain tissue D_eff_/D_free_, which is 0.43 under control (lower left plot in Fig. 1), 0.55, 5–10 min post tDCS (lower middle plot) and 0.46, 25–30 min post tDCS (lower right plot). Table 3 summarizes the values for D_free_ of sodium fluorescein, BSA (negatively charged, charge number − 19) and Dex-70k (neutral), and D_eff_/D_free_ for each solute under control, 5–10 min and 25–30 min post tDCS, respectively. Figure 2 shows the comparison of D_eff_/D_free_ for each solute under these conditions. We can see that D_eff_/D_free_ increases from 0.11 under control to 0.24 (or 0.25), 5–10 min post tDCS for the large solutes, BSA and Dex-70k, ~ 2.2-fold. However, for the small solute, sodium fluorescein, D_eff_/D_free_ increases from 0.45 to 0.50, only ~ 1.1-fold.

**Table 3:** tDCS modulated effective solute diffusion coefficients in brain tissue.

Solute	Stokes radius (nm)	D_free_ (× 10^−7^ cm^2^/s)	$\frac{{{\text{D}}_{eff control} }}{{{\text{D}}_{{{\text{free}}}} }}$	$\frac{{{\text{D}}_{eff 5 - 10\min post tDCS} }}{{{\text{D}}_{{{\text{free}}}} }}$	$\frac{{{\text{D}}_{eff 25 - 30\min post tDCS} }}{{{\text{D}}_{{{\text{free}}}} }}$
Sodium fluorescein	0.45	56.2	0.45 ± 0.03 (n = 5)	0.50 ± 0.03 (n = 5)	0.45 ± 0.01 (n = 6)
FITC-BSA (− 19)	3.5	8.83	0.11 ± 0.02 (n = 7)	0.24 ± 0.05 (n = 7)	0.12 ± 0.02 (n = 8)
FITC-Dex-70k	3.6	7.23	0.11 ± 0.03 (n = 6)	0.25 ± 0.04 (n = 7)	0.12 ± 0.01 (n = 7)

Values are mean ± SD. n = number of vessels.

## Discussion

Expanding on prior mechanisms of action of tDCS such as neuronal polarization^49–52^, enhancement in regional blood flow^9,27–30,53,54^ and in blood nitric oxide (NO) levels^31, 32^, as well as transient increase in the BBB permeability^23^, our current study uncovered a new target of tDCS: the extracellular space (ECS). By employing non-invasive high resolution (submicron) multiphoton microscopy^44^ to image solute spreading around cerebral microvessels in rat brain and fitting the spatio-temporal solute distribution profiles by an unsteady diffusion transport model, we report that tDCS transiently increases the effective solute diffusion coefficients in rat brain tissue D_eff_ to various sized solutes. The increasing level is dependent on the size of the solute but independent of the charge carried by the solute for the size of the solutes under this study.

The brain includes functional cellular elements (neurons and glial cells), transport systems including the blood vasculature and BBB, and the ECS surrounding the cellular elements and blood vasculature. Under typical conditions, about 20% of the total brain tissue is occupied by the ECS^34,36,55^. Aging as well as CNS diseases and injuries can significantly change the percentage of ECS volume in brain tissue^56,57^. ECS volume fraction can decrease by ~ 25% with late aging^34^, and in rodent models, learning deficits are correlated with decrease in ECS fraction and altered diffusion parameters^58^. In a mouse model of Alzheimer’s disease, a further ~ 50% reduction in volume fraction and ~ 5% diffusivity decrease were closely related to plaque deposition, and associated impaired navigation^57^. ECS decreases several fold (to ~ 5%) minutes after severe ischemia^59^. Altered ECS fraction and diffusivity is reported in animal models of Parkinson’s disease, epilepsy, multiple sclerosis^34,60,61^. Changes in ECS diffusion would broadly be expected in any cases of inflammation edema, or progressive neurodegeneration^34^. Despite the role of the ECS in brain function and disease, interventions to target ECS are either non-specific (e.g. osmotic stress) or not clinically translatable (e.g. genetic modification). In contrast, tDCS is safe^2,62,63^ and broadly used even on healthy subjects^64,65^.

The gaps between cells forming the ECS are ~ 38–64 nm^37,45^. The ECS is filled with negatively charged extracellular matrix (ECM) and interstitial fluid that is similar with ionic composition to the cerebrospinal fluid (CSF) that maintains an ionic microenvironment important in nerve and glial cell function as well as the vascular osmotic pressure^66,67^. Brain ECM consists of a matrix-like network formed around a backbone of hyaluronic acid (HA), a long, highly hydrated non-sulfated glycosaminoglycan, chondroitin sulfate (CS) and heparan sulfate (HS) proteoglycans along with various glycoproteins, laminins and collagens^36,38–40^. Both the cell membranes enclosing the ECS and the ECM inside the ECS contribute significant resistance to the solute transport in the ECS (brain tissue) especially to large solutes. To predict this resistance and to estimate the effective solute diffusion coefficients in the brain ECS, we used the following simplified formula, derived from rigorous theoretical models from hydrodynamics and transport phenomena and summarized in^68^. The diffusion transport of a solute is retarded by the friction between the solute and the cell membranes/ECM and by the steric hindrance due to the existence of the cell membranes and the ECM. The effective solute diffusion coefficient thus changes with the gap spacing of the ECS, ECM density/arrangements and the solute size. In a fiber matrix (ECM) without boundaries,

1 $$\frac{{D_{matrix} }}{{D_{free} }} = 1 - \left[ {\left( {1 - \varepsilon } \right)^{0.5} \left( {1 + \frac{2a}{{\pi^{0.5} r_{f} }}} \right)} \right]$$

Here, $a$ is the solute radius, *r*_*f*_ is the fiber radius, *S*_*f*_ is the volume fraction of fibers in ECM, *ε* = 1 − *S*_*f*_ is the void volume fraction. *D*_*free*_ is the solute diffusion coefficient in free aqueous solution (CSF) at 37 °C in our study^69^. In the ECS filled with ECM,

2 $$\frac{{D_{ECS} }}{{D_{free} }} = \left\{ {1 - \left[ {\left( {1 - \varepsilon } \right)^{0.5} \left( {1 + \frac{2a}{{\pi^{0.5} r_{f} }}} \right)} \right]} \right\}\left( {1 - \beta} \right)\left( {1 - 1.004\beta + 0.418\beta^{3} + 0.210\beta^{4} - 0.1696\beta^{5} } \right)$$

Here, *β* = 2*a*/W, *a* is the solute radius and W is the gap spacing of ECS.

Under control conditions, W is equivalent to ~ 40 nm as estimated in the rat brain ECS^37^. Previously, Li et al.^69^ used r_f_ = 6 nm and S_f_ = 0.326 to simulate the glycocalyx at the endothelial surface and the ECM in the basement membrane of the BBB in their transport model, which successfully predicted the measured permeability data. Since the BBB endothelial surface glycocalyx and ECM in the BM contain proteoglycans and glycosaminoglycans^70^, similar to those in the brain ECM of the ECS, we assumed the same mean radius of the fiber r_f_ = 6 nm in the brain ECM, but smaller volume fraction S_f_ = 0.17. The predicted D_eff_/D_free_ of various sized solutes in the brain tissue by Eq. (2) reconcile with that measured under control conditions. If we assumed that 5–10 min post tDCS transiently enhances the ECS gap spacing W from 40 to 60 nm (1.5-fold increase) and reduces S_f_ from 0.17 to 0.11 (1.54-fold decrease) accordingly, the predicted D_eff_/D_free_ also match the measured data. Table 4 summarizes these predictions under control and in response to tDCS. Our predictions suggest a structural mechanism by which tDCS modulates the solute transport in brain tissue.

**Table 4:** Model predictions for effective solute diffusion coefficients in brain tissue.

Solute	Stokes radius (nm)	$\frac{{{\text{D}}_{eff control} }}{{{\text{D}}_{{{\text{free}}}} }}$ (W = 40 nm, S_f_ = 0.17)	$\frac{{{\text{D}}_{eff 5 - 10\min post tDCS} }}{{{\text{D}}_{{{\text{free}}}} }}$ (W = 60 nm, S_f_ = 0.11)
Sodium fluorescein	0.45	0.441	0.512
FITC-BSA (− 19)	3.5	0.124	0.254
FITC-Dex-70k	3.6	0.117	0.247

W, gap spacing in brain extracellular space (ECS); S_f_, volume fraction of fibers in ECM.

Because the ECM carries negative charge, it should induce different resistance to the transport of neutral and charged molecules. However, our measured D_eff_/D_free_ for the similar sized neutral (Dex-70k) and negatively charged BSA (− 19) were not significantly different either under control or in response to tDCS, although the BBB permeability to BSA is significantly smaller than that to Dex-70k under control since endothelial surface glycocalyx and ECM in the basement membrane of the BBB carry negative charges^48^. One possible explanation for this is that the charge density in the ECM of ECS is smaller than that in the endothelial surface glycocalyx and basement membrane of the BBB due to a smaller fiber matrix density S_f_. The resistance from the steric hindrance and friction is much greater than that from the electrostatic exclusion for these sized molecules.

Transport of solutes are by two mechanisms: diffusion and convection. Diffusion is determined by the solute diffusivity, which is dependent on the solute size, shape, charge, the porosity of the tissue, e.g., available volume in the ECS (interstitial space), and the viscosity of the solution in the interstitial space, temperature, etc. and the driving force, which is the concentration gradient. Convection is the solute transport carried by the fluid flow, which is dependent on the fluid (e.g. CSF) flow velocity and the solute concentration. The driving force for the fluid transport is the pressure gradient. In Xie et al.^71^ and others, they used fluorescent tracers (solutes) to indicate the transport of CSF, which only represents the convection transport of the solutes. However, if the resistance of brain tissue decreases (e.g., ECS increases) to the fluid transport (e.g. CSF transport increases), it should also decrease to the diffusion transport of a solute (solute diffusivity increases) under the same driving forces (e.g. concentration and pressure gradients). Our findings that tDCS enhances solute diffusivity in the brain tissue by modulating the ECS are consistent with theirs. According to Xie et al.^71^, adrenergic signaling not only plays an important role in modulating neuronal activity but also the volume of the interstitial space (ECS). Monai et al.^20^ also reported that tDCS-induced elevation in astrocytic Ca^2+^ is dependent on alpha-1 adrenergic receptor. Based on their studies, it is suggested that tDCS modulates the solute brain transport and ECS, as well as the BBB permeability, possibly through an adrenergic signaling pathway.

In conclusion, we report here that in addition to increasing the BBB permeability, tDCS transiently increases the solute transport in the brain tissue, suggesting a new motion of action of tDCS targeting the ECS by increasing its gap spacing. These findings imply that tDCS can change the microenvironment surrounding neurons, glial cells and vasculature to achieve its therapeutic effects – though such links remain to be established. Given the universal role of ECS in brain function, this motion of action complementary to standard neurophysiological outcomes of tDCS including altered excitability^49,72,73^ and synaptic plasticity^14,19,74,75^, as well as reported morphological and molecular changes^76,77^. Finally, these findings also suggest that tDCS can be applied to enhance the drug brain delivery, especially macromolecules, through the non-invasive route from the BBB and the minimum-invasive route by CSF if injected through brain ventricles and spinal cord.

## Methods

### Animal preparation

**Figure 3:**
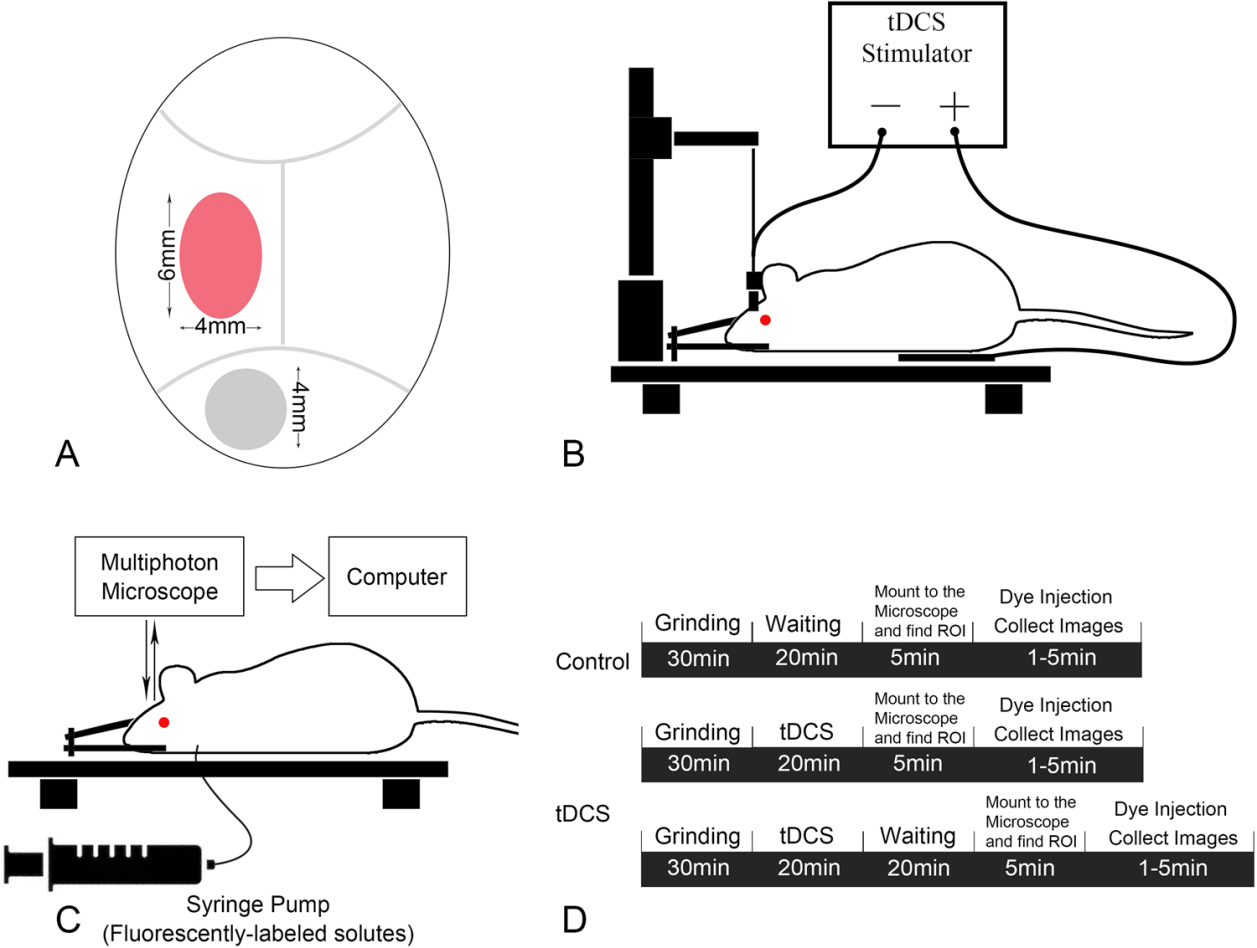
Experimental set-ups, timelines and protocols. (**A**) Illustration showing the locations for the tDCS treatment and imaging on the rat skull. The grey circled region (~ 4 mm diameter) is for the tDCS application. The red elliptical area (~ 6 mm × 4 mm) indicates the imaging region with the thinned skull. (**B**) Sketch for the tDCS application to the rat head. One electrode (−) connects to the rat skull and another electrode ( +) connects to the thoracic area. (**C**) Set-ups for determining the BBB solute permeability (P) and brain tissue diffusion coefficient (D_eff_) by multiphoton microscopy. Through the imaging region shown by the red ellipse in (A), the ROI containing the microvessel and surrounding tissue is focused in the brain parenchyma ~ 100–200 μm below the pia mater. The images for the ROI are collected simultaneously when the solution with the test solutes is injected via the carotid artery. P and D_eff_ are determined by analyzing the collected images (see Fig. 4) off-line. (**D**) Sketch for the protocols and timelines for animal skull preparation and image collections for the P and D_eff_ measurement under control and after tDCS.

All experiments were performed on adult female Sprague–Dawley rats (250–300 g, 3–4 months), supplied by Hilltop Laboratory Animals (Scottdale, PA). The Institutional Animal Care and Use Committee (IACUC) at the City College of the City University of New York approved the animal care and preparation procedures. All experiments were performed in accordance with relevant guidelines and regulations (The protocol number is 964). Rats were anesthetized with sodium pentobarbital injected subcutaneously. The initial dose was 65 mg/kg bodyweight. The depth of anesthesia was monitored for the absence of withdrawal reflex to toe pinch and absence of blink reflex. Anesthesia was further checked every 15 min during the experiment and the rat was given an additional dose of 3 mg/dose as needed. A heating pad was used to keep rat’s body temperature in the experiment. When the experiment was done, the animal was euthanized by intravenous injection of an overdose of pentobarbital (> 100 mg/kg). The preparation of the rat skull observation area was the same as described in previous studies^44,78,79^. In short, after anesthetized, the region of interest (ROI) on the rat skull was exposed by removing the hair, skin and connective tissue. A ~ 6 mm by ~ 4 mm section (ROI) on the right or left frontoparietal bone (Fig. 3A) was ground with a high-speed micro-grinder (0–50,000 rpm, DLT 50KBU, Brasseler USA, Savannah, GA) until a part of it (~ 2 mm × 2 mm) became translucent. In the process, the artificial cerebrospinal fluid (ACSF) at the room temperature was applied to the surface of the skull to remove the heat generated by grinding. After grinding, the carotid artery on the same side of the ROI was cannulated with a PE50 tubing (BD Medical, NJ). The rat was then placed on a stereotaxic alignment system (SAS 597, David Kopf Instruments, Tujunga, CA), and its head was fixed with two ear bars and a mouth clamp. After tDCS treatment, the cross-sectional images of a cerebral microvessel and its surrounding brain tissue were observed and collected by a multiphoton microscope through the translucent part of the skull. The BBB solute permeability and solute diffusion coefficient in the brain tissue were determined off-line from the collected images. Only one experimental condition and 1–2 vessels can be collected in each rat.

**Figure 4:**
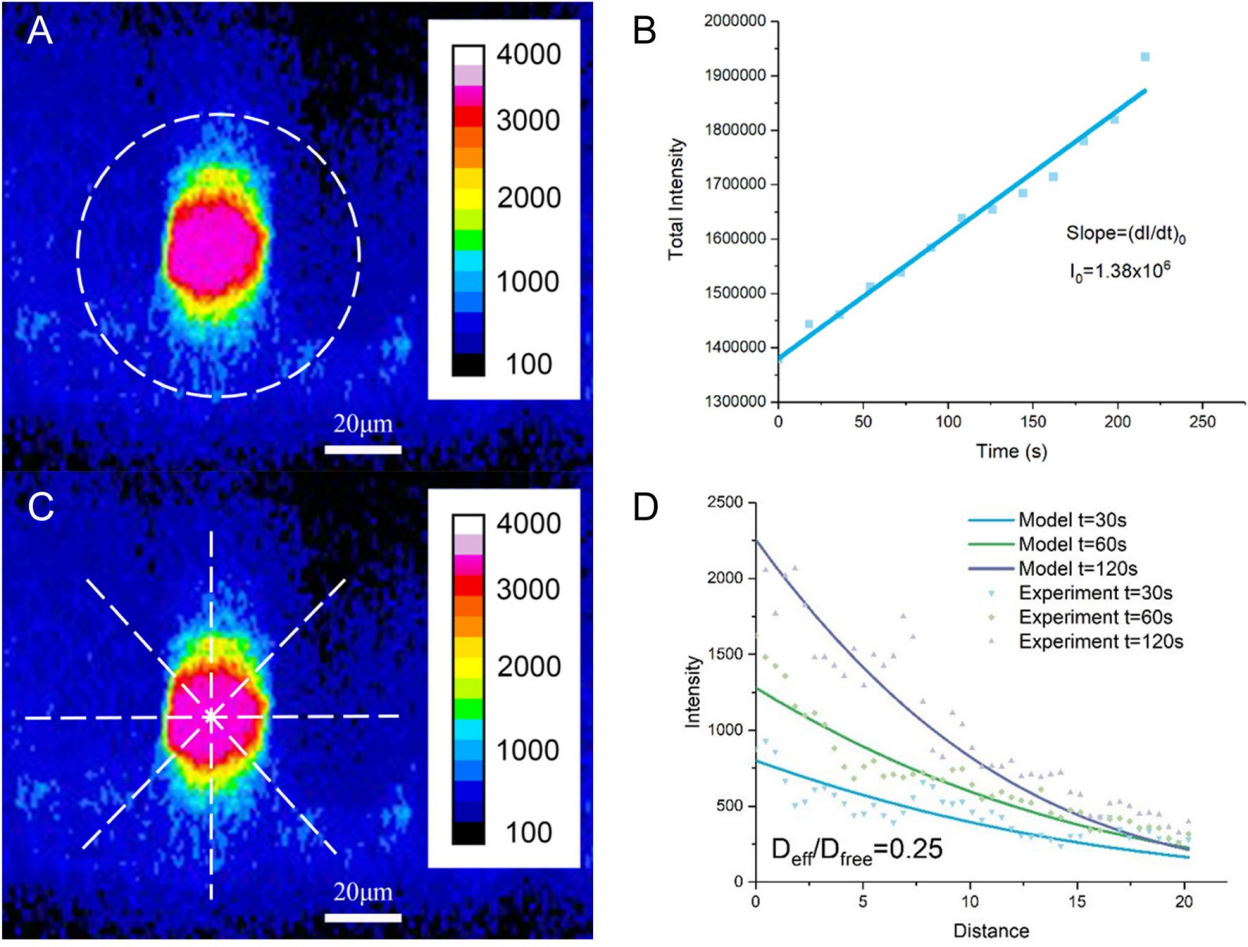
Methods for determining BBB solute permeability (P) and solute diffusion coefficient in rat brain tissue (D_eff_). (**A**) A cross-sectional image showing a cerebral microvessel filled with fluorescent solutes and the surrounding tissue. The dashed line enclosed region is the ROI for determining P. The edge of the dashed line is about 10–30 μm far from the vessel wall to prevent contamination from the neighboring microvessels. The ROI should be enough large to include the spreading dye from the lumen of the vessel during image collection for the P measurement. (**B**) Curve for total intensity of the test solutes in the ROI vs. perfusion time. The slope of the curve (dI/dt)_0_ during the initial solute accumulation period is used for determining P. P = 1/I_0_ *(dI/dt)_0_* r/2. I_0_ is total fluorescence intensity in the vessel lumen and r is the vessel radius. (**C**) From the cross-sectional image of the ROI, radial lines in 8 directions are drawn from the vessel center. (**D**) The intensity averaged from the eight radial lines is plotted from the vessel wall at different times (t = 30, 60 and 120 s) (dotted lines). The solid smooth lines are the best fitting curves of the model prediction at the corresponding times when the correct effective solute diffusion coefficient D_eff_ is chosen. D_eff_/D_free_ = 0.25 (D_free_ is the free solute diffusion coefficient in an aqueous solution at 37 °C) is the best fitting value for this run of the experiment for FITC-BSA 5–10 min post tDCS.

### Solutions and fluorescent test solutes

#### Mammalian Ringer’s solution

Mammalian Ringer’s solution was used for all perfusates, which is composed of (in mM) NaCl 132, KCl 4.6, MgSO_4_ 1.2, CaCl_2_ 2.0, NaHCO_3_ 5.0, glucose 5.5, and HEPES 20. The pH was buffered to 7.40–7.45 by adjusting the ratio of HEPES acid to base. In addition, the florescent dye solution contained 10 mg/mL BSA (A4378; Sigma-Aldrich, USA)^23,44,79^. The solutions were made fresh on the day of use to avoid binding to the serum albumin.

#### Artificial cerebrospinal fluid (ACSF)

The ACSF solution composition is (in mM) NaCl 110.5, KCl 4.7, CaCl_2_ 2.5, KH_2_PO_4_ 1.1, MgSO_4_⋅7H_2_O 1.25, NaHCO_3_ 25, and HEPES. The solution was buffered to pH 7.4 ± 0.5^23,44,79^. All chemicals were purchased from Sigma-Aldrich (USA).

#### *Sodium fluorescein, FITC-BSA and FITC-dextran-*70k

Sodium fluorescein (F6377, Sigma-Aldrich; mol. wt. 376 Da, Stokes radius ~ 0.45 nm) was dissolved at 0.1 mg/mL in the Ringer solution containing 10 mg/mL BSA. FITC-BSA (A9771, Sigma-Aldrich; mol. wt. ~ 69,000 Da, Stokes radius ~ 3.5 nm) and FITC-dextran-70kD (FD70s, Sigma-Aldrich; mol. wt. 70,000 Da, Stokes radius ~ 3.6 nm) was at the concentration of 1 mg/mL in the Ringer solution containing 10 mg/mL BSA^44^. The concentration of the solution for each solute was chosen to be in the linear range of the concentration vs. fluorescence intensity calibrated in Shi et al.^44^.

### Transcranial direct current stimulation (tDCS)

A constant current stimulator (1 × 1 tDCS, Soterix Medical Inc, New York, USA) was used to deliver a 1 mA current for 20 min for the tDCS as previously described^32,80,81^. To obtain similar physiological outcomes as in the human tDCS application studies^32^, the current was applied transcranially to the frontal cortex of a rat head (approximately 2 mm anterior to Bregma and 2 mm right to Sagittal suture) (Fig. 3A). Specifically, an epicranial anode electrode (1 mm diameter, Ag/AgCl) inside a 3D-printed electrode holder (contact area = 12.56 mm^2^) was positioned onto the skull (a round area with ~ 4 mm diameter shown in Fig. 3A). A conductive hydrogel uniformly filled the electrode holder (Signa, NJ, USA). A rotating adjustable clamp and a precise micromanipulator from Narishige International USA, Inc. (NY, USA) was used to secure the electrode and the holder in place over the stimulation area. The returning electrode (5 × 5 cm adhesive conductive fabric electrode) from AxelGaard Manufacturing Co., Ltd. (CA, USA) was placed onto the ventral thoracic region of the rat with hair removed (Fig. 3B) and a thin layer of Signa gel was spread to maintain uniform skin–electrode contact. Because the experimental preparation separates the brain region imaged from that directly under the stimulating electrode^23,81^, responses measured here are from brain regions with reduced current density.

### Experimental protocol

After tDCS, the rat head was immediately positioned under the multiphoton microscope (Fig. 3C) for the measurement of BBB solute permeability (P) and solute diffusion coefficients in the brain tissue (D_eff_). Figure 3D summarizes the experimental protocols for the control and tDCS. It took about 5 min to mount the rat head to the multiphoton microscope and find the ROI. Therefore, the images for the measurement were first collected ~ 5 min post-tDCS. Our recent study showed that the BBB permeability transiently increased by tDCS until 20–25 min post 20 min–1 mA tDCS^23^. We thus collected the images ~ 5 and 25 min post-tDCS and determined the D_eff_ from analyzing these collected images.

### Multiphoton microscopy and image collection

An Ultima Multiphoton Microscopy system (Prairie Tech., Inc., WI, USA) was used to collect 12-bit images in vivo. The excitation wavelength was set to 820 nm for the solutes used in the current study and a water immersion lens (40 × /NA0.8) was used to observe the microvessels about 100–200 microns below the pia mater^44^. A syringe pump injected the solution with fluorescently labeled solutes at a constant rate of ~ 3 ml/min into the cerebral circulation. 3 ml/min is the normal blood flow rate at the rat carotid artery^79^. The dye took about 10–15 s from the cannulation site at carotid artery to the cerebral microvessels. While the dye was introduced into the cerebral circulation from the carotid artery, the images were taken simultaneously. The post-capillary venules of 20–40 µm diameter^23,44,79^ were selected in a ROI with a volume of ~ 200 µm × 8 µm × 100 µm (x, y, z) and the images were collected at a rate of 5–15 s/image. The spatial resolution of an image is ~ 0.47 µm × 0.47 µm × 1 µm in x, y and z directions. The collected images were then transferred to an image acquisition and analysis workstation for off-line determination of P and D_eff_.

### Image analysis

The Image J (National Institutes of Health) was used to analyze the collected images. First the images were reconstructed into a segment of 200 µm × 100 µm cross-sectional area (x–z) with 8 µm thickness. The temporal and spatial solute intensity (concentration) profiles I (t, x, z) surrounding a microvessel in this volume of the brain tissue were determined by the ImageJ program.

### Determination of the BBB solute permeability P and effective solute diffusion coefficient D_eff_ in brain tissue

The same method as in our previous study was used to determine the permeability (P) of the cerebral microvessels and effective solute diffusion coefficient (D_eff_) in brain tissue^23,44^. A cross-sectional image (x–z) of a rat cerebral microvessel filled with a solution of fluorescently-labeled solutes and the surrounding brain tissue was illustrated in Fig. 4A. The white dashed line circled region is the ROI to determine P. The caption for Fig. 4B describes how to determine the BBB solute permeability P. Figure 4C,D illustrate how to determine D_eff_ from the collected images. *D*_*eff*_ was determined by fitting the temporal and spatial intensity curves by an unsteady mathematical model for solute transport in the tissue space^82^

3 $$\frac{{\partial C_{t} }}{\partial t} = {\text{D}}_{{{\text{eff}}}} \left( {\frac{{\partial^{2} C_{t} }}{{\partial r^{2} }} + \frac{1}{r}\frac{{\partial C_{t} }}{\partial r}} \right) - {\chi u}\frac{{\partial C_{t} }}{\partial r}$$

where C_t_ (t, r) is the concentration of solutes in the tissue space, D_eff_ is the effective diffusion coefficient of solutes in tissue, *r* is the distance from the vessel center. *χ* is the retardation coefficient of a solute in the tissue, estimated as 0.1–1 for solutes under study^47^. u is the interstitial fluid velocity in brain tissue. The Peclet number P_et_ in the tissue is^83^,

4 $$P_{et} = \frac{{{\upchi }VL_{t} }}{{{\text{D}}_{{{\text{eff}}}} }}$$

Here L_t_ is the characteristic length for the solute tissue transport, which is the mean half distance (~ 20 μm) between adjacent microvessels^84^, *V* is the characteristic interstitial fluid velocity, which is approximated by the outflow velocity from the vessel wall L_p_Δp_eff_. L_p_ is the hydraulic conductivity of the microvessel, ~ 2 × 10^−9^ cm/s/cm H_2_O^85^, while Δp_eff_ is the effective pressure difference across the vessel wall, which is less than 10 cm H_2_O^79^. For the size range of solutes in this study, D_eff_ is in the range of 10^−6^ ~ 10^−8^ cm^2^/s, P_et_ was calculated as in the order of 10^−5^–10^−2^ even assuming that the L_p_ increases by 100 folds due to tDCS treatment. Due to the very low Peclet number, the convection part can be neglected in Eq. (3). Equation (3) becomes,

5 $$\frac{{\partial {\text{C}}_{{\text{t}}} }}{{\partial {\text{t}}}} = {\text{D}}_{{{\text{eff}}}} \left( {\frac{{\partial^{2} {\text{C}}_{{\text{t}}} }}{{\partial {\text{r}}^{2} }} + \frac{1}{{\text{r}}}\frac{{\partial {\text{C}}_{{\text{t}}} }}{{\partial {\text{r}}}}} \right)$$

The boundary conditions for Eq. (5) are,

6 $${\text{at the vessel wall r}} = {\text{a}},\;\;{\text{P}}\left( {{\text{C}}_{{{\text{lumen}}}} - {\text{C}}_{{\text{t}}} } \right) = {\text{D}}_{{{\text{eff}}}} \frac{{\partial {\text{C}}_{{\text{t}}} }}{{\partial {\text{r}}}}$$

7 $${\text{midway between adjacent vessels r}} = {\text{b}},\;\;\frac{{\partial {\text{C}}_{{\text{t}}} }}{{\partial {\text{r}}}} = 0$$

8 $${\text{The initial condition is}},\;\;{\text{t}} = 0,\;{\text{C}}_{{\text{t}}} (0,{\text{r}}) = 0$$

where C_lumen_ is the solute concentration in the vessel lumen, P is the microvessel solute permeability. Both of which can be determined from the collected images. The only unknown parameter in Eqs. (5)–(8) is D_eff_. Solving above Eq. (5) with an assumed value of D_eff_ by Matlab, we obtained the theoretical solute tissue concentration profiles C_t_(t, r_t_). To obtain the measured C_t_(t, r_t_), eight straight lines were drawn from the center of a vessel lumen to a distance ~ 20 µm from the vessel wall in the tissue space (Fig. 4C), the averaged intensity from these 8 directions was approximated as the measured C_t_(t, r_t_), which was plotted in Fig. 4D (colored dots, distance *r*_*t*_ = 0 is at the vessel wall). The D_eff_ was determined by the best curve-fitting of the model predictions (colored lines in Fig. 4D) to the measured profiles.

### Influence of red blood cells (RBCs), free dye, and solvent drag on BBB permeability

While the dye solution was injected at the perfusion rate of 3 ml/min, the normal blood flow rate at the rat carotid artery^86,87^, there was still residue blood (red blood cells, RBCs) in the cerebral microvessels. As estimated in Yuan et al.^79^, this residue blood overestimates the measured BBB permeability P^measure^ by ~ 11%, Besides RBCs, free dye overestimates the permeability to the solutes labeled with the fluorescent dye^79,88^. The solute permeability affected by the free dye was estimated by P^correct^ = [1/(1 − F)] P^measure^ − [F/(1 − F)] P^freedye^^88^, where P^correct^ is the corrected permeability; P^freedye^ is the permeability to the free dye. Because the size of sodium fluorescein (NaFl, 376) is similar to that of FITC (389.4), P^freedye^ ~ P^NaFl^; F is the intensity ratio of the free dye filtrate to the fluorescently labeled solution. F is ~ 0.1% for the FITC-labeled solutes in the current study; The above corrected P still overestimates the true diffusive solute permeability P_d_ due to the solvent drag coupled to the fluid flow. The following equations were used to find the P_d_ of the solutes under this study^88,89^,

9 $$P = P_{d} \frac{Pe}{{{\exp}\left( {Pe - 1} \right)}} + L_{p} \left( {1 - \sigma } \right)\Delta p_{eff}$$

10 $$P_{e} = \frac{{L_{p} \left( {1 - \sigma } \right)\Delta p_{eff} }}{{P_{d} }}$$

where L_p_, the hydraulic conductivity of the microvessel, is ~ 2.0 × 10^−9^ cm/s/cm H_2_O for the cerebral microvessels^44, 85^, *P*_*e*_ is the Peclet number. The reflection coefficient of the microvessel to the solute is *σ* and the effective filtration pressure Δ*p*_*eff*_ across the microvessel wall is calculated from

11 $$\Delta p_{eff} = \Delta p - \sigma^{albumin} \Delta \pi^{albumin} - \sigma^{{dye{ - }solute}} \Delta \pi^{{dye{ - }solute}}$$

Here the hydrostatic pressure difference across the cerebral microvessel wall Δ*p* was ~ 10 cm H_2_O, and the osmotic pressure difference Δ*π*^albumin^ was 3.6 cm H_2_O for 1% BSA^79^. The superscript dye-solute is FITC-BSA, Dex-70k or sodium fluorescein. Based on the previous studies^79^, σ of rat cerebral microvessels to the test solutes were estimated as 0.95 and 0.1, respectively, for σ^dextran−70k^ (the same as σ^albumin^) and σ^NaFl^. In correcting influence from the solvent drag in the permeability, for the control group and 25–30 min post tDCS group, L_p,control_ = 2 × 10^−9^ cm/s/cm H_2_O; for the 5–10 min post tDCS group, 100 × L_p,control_ was used.

### Data analysis and statistics

Data are presented as means ± SE. The control P was the average value of the permeability measured under control. This control P value was used to normalize all the subsequent treatments correspondingly. D_eff_ was given as D_eff_/D_free_. Here, D_free_ is the free solute diffusion coefficient in water at 37 °C. The statistical significance was determined by applying ANOVA to the treatment at different times and to between-group data for the differences at specific times. *p* < 0.05 was considered statistically significant.
